# Generating carbon finance through avoided deforestation and its potential to create climatic, conservation and human development benefits

**DOI:** 10.1098/rstb.2007.0029

**Published:** 2008-02-11

**Authors:** Johannes Ebeling, Maï Yasué

**Affiliations:** 1 EcoSecurities40/41 Park End Street, Oxford OX1 1JD, UK; 2 Project Seahorse, Fisheries Centre, University of British ColumbiaVancouver, BC, Canada V6T 1Z4

**Keywords:** biodiversity, carbon market, climate change, governance, Kyoto Protocol, rainforest

## Abstract

Recent proposals to compensate developing countries for reducing emissions from deforestation (RED) under forthcoming climate change mitigation regimes are receiving increasing attention. Here we demonstrate that if RED credits were traded on international carbon markets, even moderate decreases in deforestation rates could generate billions of Euros annually for tropical forest conservation. We also discuss the main challenges for a RED mechanism that delivers real climatic benefits. These include providing sufficient incentives while only rewarding deforestation reductions beyond business-as-usual scenarios, addressing risks arising from forest degradation and international leakage, and ensuring permanence of emission reductions. Governance may become a formidable challenge for RED because some countries with the highest RED potentials score poorly on governance indices. In addition to climate mitigation, RED funds could help achieve substantial co-benefits for biodiversity conservation and human development. However, this will probably require targeted additional support because the highest biodiversity threats and human development needs may exist in countries that have limited income potentials from RED. In conclusion, how successfully a market-based RED mechanism can contribute to climate change mitigation, conservation and development will strongly depend on accompanying measures and carefully designed incentive structures involving governments, business, as well as the conservation and development communities.

## 1. Introduction

Deforestation in the tropics accounts for up to 20% of global emissions of carbon dioxide (CO_2_), making it the second most important contributor to climate change after the combustion of fossil fuels and the largest source of greenhouse gas (GHG) emissions in the developing world (Houghton 2005). Despite this, the current framework under the United Nations Framework Convention on Climate Change (UNFCCC) and its Kyoto Protocol does not contain any mechanism to reward efforts aimed at avoided deforestation in developing countries. At present, the possibilities to gain carbon credits from forestry activities under the clean development mechanism (CDM) remain limited to afforestation and reforestation projects (UNFCCC 2001).

The debate has recently gained new momentum, however, with proposals to compensate developing countries that succeed in reducing emissions from deforestation (RED) with financial incentives, such as tradable carbon credits (Laurance 2007). The concept would most likely involve countries lowering deforestation rates below a national historical baseline, and a novel mechanism could be included in a post-2012 Kyoto regime (Santilli *et al*. 2005; UNFCCC 2005*a*). Crediting emission reductions on a national rather than on a project level would have the major advantage of accounting for in-country ‘carbon leakage’ which occurs when deforesting activities are simply displaced rather than avoided (Aukland *et al*. 2003). RED would thereby address one of the major objections raised in past policy discussions against including avoided deforestation under the CDM.

Globally, the Amazon stands out as the region with the greatest potential to contribute to climate change mitigation through RED. Between 1990 and 2005, all Amazon countries combined accounted for approximately 26% (3.7 Mha) of global deforestation (table 1; FAO 2001, 2006). Moreover, high average forest carbon densities make this figure translate into 46% of related global carbon emissions. For example, Brazil ranks among the world's top GHG emitters worldwide when emissions from deforestation are taken into account (UNFCCC 2005*b*).

Reducing deforestation would significantly reduce global GHG emissions and markets for RED carbon credits could be substantial. However, in order to translate this potential into actual climate benefits, several critical issues must be addressed by a future policy framework. This paper evaluates the potential of RED to generate funds through international carbon markets and discusses four key issues that are currently debated by scientists and policy makers. In addition, we explore to what extent RED may generate biodiversity and development co-benefits. Technical questions regarding carbon measurements and the monitoring of land-use change will not be discussed, since there is a general scientific consensus that these can be resolved pragmatically with existing technology and that uncertainties can be addressed through conservative carbon accounting approaches.

## 2. Carbon finance for avoided deforestation

Linking RED to international carbon markets could create a real opportunity to tackle an important source of GHG emissions at comparably low costs and could overcome the funding constraints that have hampered forest conservation for many years (Balmford & Whitten 2003; Laurance 2007). International funding for forestry totalled approximately US$1.1 billion annually during the last decade with expenditures for forest protection probably being much lower (Tomaselli 2006). In comparison, international carbon markets transacted €9.5 billion (US$14.1 billion) in 2005, the year when the Kyoto Protocol entered into force, a figure that soared to €22.5 billion (US$33.3 billion) in 2006 and is projected to continue to increase (Hasselknippe & Røine 2007).

**Figure 1:**
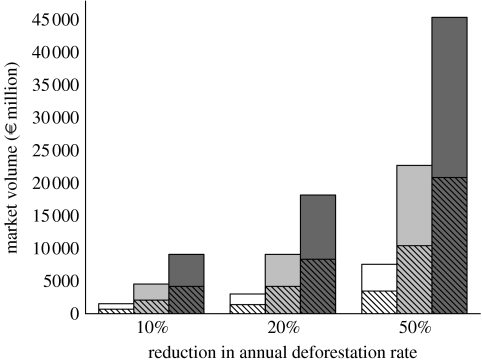
Scenarios for potential global market value of RED credits at variable carbon prices and reductions in deforestation rates. Bars display global potential market value, and diagonal lines represent the contributions of Amazon countries. Carbon price €/tCO_2_: open bars, 5; grey bars, 15 and black bars, 30.

To evaluate the potential scale of conservation carbon finance created through RED, we computed a range of estimates using existing reported data on annual deforestation rates between 1990 and 2005 for all countries with a net forest area loss during this period (FAO 2006) and country-specific values of average forest carbon content (IPCC 2003). We then factored in potential decreases in deforestation rates and a range of carbon prices (€5–30/tCO_2_) recently observed on international markets. Using these assumptions, reducing deforestation rates by as little as 10% globally could generate substantial annual carbon finance (€1.5–9.1 billion, or US$2.2–13.5 billion; figure 1).

## 3. Setting fair and realistic baselines

Under a future RED scheme, carbon credits would be created based on a comparison of current deforestation rates and a business-as-usual (BAU) or ‘baseline’ scenario. This gives the setting of baselines a crucial role in defining both the monetary incentives for individual countries and the environmental integrity of generated carbon credits. In the discussions to date, the favoured approach has been to define baselines as a multi-year average of historical deforestation rates. This approach would favour countries with high historical deforestation rates, for example, Brazil or Ecuador. In contrast, countries which have maintained a very high forest cover, for example, Guyana or Suriname, or have already succeeded in lowering or even reversing deforestation trends in the past, for example, Chile or Costa Rica, would gain very little from RED.

Such seemingly inequitable approaches to calculating baselines could jeopardize political support for RED from countries with a good deforestation track record. In addition, incorrectly assuming deforestation rates to remain high while they are in fact improving under the BAU scenario would create ‘hot air’ by rewarding reductions in GHG emissions that would have occurred anyway. A number of countries demonstrate ‘forest transitions’ (Rudel *et al*. 2005), trends of decreasing deforestation rates over time, which may eventually reverse into increases in forest cover, owing to the shifts in labour markets and demands for environmental services from forests associated with economic development. On the other end of the spectrum, in some countries, low historical deforestation rates could rapidly change under a realistic BAU scenario as land-use conversion pressures increase with economic and demographic growth. In this case, RED schemes could fail to provide sufficient economic incentives to prevent emission increases. It is noteworthy that similar challenges existed in negotiating baselines for the current Kyoto Protocol, for example, regarding vastly different energy efficiency levels in the USA and Japan, or the BAU decrease in emissions through the economic collapse in East Germany (Depledge 2000).

## 4. Accounting for emissions from degradation

To measure and monitor deforestation, countries must agree on a threshold value distinguishing forests from non-forest land. Under the Kyoto Protocol, forests are defined as lands with more than 10–30% crown cover. However, many activities such as selective logging can degrade forests without causing significant changes to crown cover that could be detected by most types of remote sensing technology (DeFries *et al*. 2005). Consequently, even if there are no net changes in forested area, there could be significant carbon losses. Asner *et al*. (2005) estimated that emissions from land-use conversions in the Amazon are 25% greater when forest degradation is included. Degradation could thus undermine the climatic benefits of RED while furthermore threatening biodiversity.

Many scientists have therefore argued for a mechanism aimed at reducing emissions from deforestation and degradation (REDD). Unfortunately, while most experts agree that forest area changes can be monitored sufficiently well with existing technology, it is much more challenging to estimate carbon stock changes from forest degradation (DeFries *et al*. 2007). However, rather than delaying the start of a scheme until suitable technologies are developed and deployed, pragmatic approaches can be implemented. For example, Achard *et al*. (2005) propose to simply discount the carbon values of non-intact forests when issuing REDD credits. Depending on available data for a country, a variable number of categories could be used to account for the severity of degradation and to assign respective REDD discounts. In addition, significant advances in remote sensing technology are underway (DeFries *et al*. 2007; Kintisch 2007) and targeted support for research and development, as well as technology transfer to REDD host countries could soon allow for more accurate measurements of carbon losses from forest degradation.

## 5. Permanence of emission reductions

Permanence of emission reductions was a major controversial issue during earlier climate negotiations regarding the inclusion of forests as carbon sinks in the Kyoto Protocol. The concern was that if a newly created sink is burnt or logged, the sequestered carbon will be released back into the atmosphere and there will be no net emission reduction (Ebeling in press). In contrast, emissions reduced by implementing a fuel switch or an energy efficiency measure will have a permanent impact. For example, even if a solar power plant goes out of service and an old oil-fired power station is reinstated, the emissions that have been avoided by the solar power plant will not become undone (Chomitz 2002).

**Figure 2:**
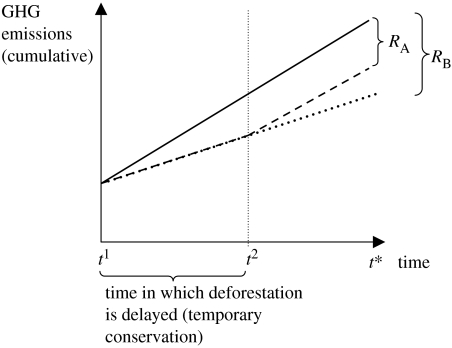
Cumulative effect of temporary and permanent emission abatement. Over time, additional emissions increase the atmospheric concentration of GHGs. The solid black line represents the BAU scenario, whereas the dotted lines represent an abatement scenario where annual emissions are reduced through activities such as forest conservation at *t*^1^. Even if the mitigation measure ends at *t*^2^, there is a lasting reduction (*R*_A_) in the amount of emissions at t^*^, albeit smaller than if the abatement measure had continued (*R*_B_).

When comparing the non-permanence issue of forest plantations to avoided deforestation, there is one crucial difference. Conserving forests decreases a source of emissions, rather than creating a sink. Emissions from deforestation are thus not inherently different from emissions created through the combustion of fossil fuels, at least in the context of large carbon pools, such as the Amazon forest or remaining global oil reserves. Protecting carbon stocks in existing forests now can mean that the stored CO_2_ is emitted later, thereby merely delaying emissions from a defined source—but the same argument can be made for fossil fuels. In fact, a time delay in emissions through temporary abatement measures results in permanent climate benefits because the cumulative atmospheric concentrations of GHGs will be lower at any future point in time (figure 2).

There is an additional benefit of temporary forest protection measures if they coincide with national level forest transitions. In countries with decreasing deforestation rates forests which are conserved through ‘temporary’ conservation measures could face lower conversion pressure once these measures terminate and could remain permanently conserved. Some concerns remain about the possibility of large deforestation ‘rebounds’ above previous levels if countries abandoned RED policies (Laurance 2007), although this scenario may be rather hypothetical (Ebeling 2006). Mechanisms such as banking a percentage of RED credits as an assurance for future deforestation increases could accommodate this concern. In contrast, any legally binding commitments resulting from RED crediting to maintain certain land areas under forest cover in perpetuity or lock in deforestation rates for the longer-term future are likely to be perceived by some countries as an infringement of sovereignty over their natural resources (Laurance 2007).

Another type of permanence risk could arise from a positive climatic feedback effect. One study predicted high temperature rises and precipitation decreases across the Amazon region (Cox *et al*. 2000), engendering fears of a massive forest ‘die-back’ due to increased drought and fire risks. However, modelling uncertainties of this and similar studies are substantial, not least because results depend on hardly predictable human activities and complex feedback loops. The Amazon has been remarkably resilient to past rapid climate change, and lowland Amazon forest cover remained largely intact even during Pleistocenic glacial periods (Colinvaux *et al*. 2000). Furthermore, predicted regional climatic changes in the Amazon region are likely to be patchy and not unidirectional, making a similarly variable ecological response probable. A number of papers in this volume allow further assessment of the likelihood of dieback (Betts *et al.* 2008).

## 6. International leakage

If deforestation activities move from one region to another within the same country, this type of ‘within-country leakage’ would be reflected in the same RED accounting system at the national level and thus become irrelevant in terms of international carbon crediting. This is one of the main advantages of using national-level instead of project-level RED mechanisms. However, a certain potential for international leakage exists. If only some countries participated in a regime for reducing deforestation, global markets might shift supply and demand patterns for timber or agricultural commodities across borders and lead to greater deforestation rates in countries not attempting to gain RED credits (Ebeling in press). During the design of the Kyoto architecture, similar concerns were voiced regarding incentives for fossil-fuel based industrial production to move from industrialized countries with emission reduction targets to (developing) countries without targets (Niesten *et al*. 2002).

Nevertheless, fears of massive international leakage in the RED context seem exaggerated. Reducing deforestation does not mean a complete halt of forest conversion. Countries trying to lower deforestation rates would first target low-cost conservation options such as clearing of forests on marginally productive soils with low opportunity costs. Governments could reduce deforestation rates in these areas by, for example, enforcing existing land regulations, especially in frontier areas, removing perverse incentives for land clearing to establish tenure, and by expanding and enforcing protected areas and indigenous territories. In many circumstances, intelligent infrastructure and land-use planning, as well as improved agricultural productivity, for example, regarding road construction and cattle farming, would greatly lower deforestation pressures (Nepstad *et al*. 2002; Soares-Filho *et al*. 2006). Thus, it is probable that highly profitable ventures, such as soya agriculture, palm oil production and logging for valuable timber, would not be strongly affected. These activities frequently involve internationally mobile actors and international leakage might indeed occur if countries were to pursue very ambitious RED targets.

A thorough understanding of the proximate and ultimate drivers of deforestation in a region or country (Lambin & Geist 2003) can help estimate the risk of cross-boundary leakage, and a comprehensive RED architecture could support leakage prevention activities in neighbouring countries. Tackling the drivers of deforestation in a sustainable manner could not only reduce leakage risks but would also provide an insurance against future rebounds of deforestation rates and thereby generate a double benefit for RED host countries and the global climate. While international leakage risks can thus be minimized, only full participation by all tropical forest countries in a RED regime would completely eliminate them, a fact that also applies to the broader emission reductions regime under Kyoto and the UNFCCC.

## 7. Governance challenge

The potential of RED to contribute to climate change mitigation, achieve co-benefits, and realize monetary benefits will ultimately depend on how successful countries actually are in lowering deforestation. This, in turn, will be influenced by their ability to, for example, enforce land-use regulations, improve road planning, implement payments for ecosystem service (PES) schemes and restructure incentives for agriculture, that is, by their governance capacity.

**Figure 3:**
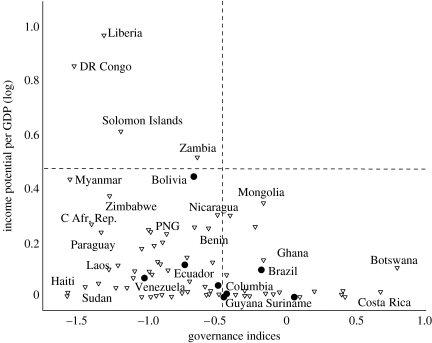
Relationship between income potential (expressed as percentage of GDP, log transformed) and governance. Governance index used is the mean of two variables measuring law enforcement and corruption perception. Lower values indicate more severe governance problems (Kaufmann *et al*. 2005). Filled circle, Amazon countries; triangle, non-Amazon countries with past net deforestation. None of the countries are located in the top right quadrant of the figure where high income potential would coincide with governance levels potentially needed to implement RED schemes effectively.

Figure 3 illustrates the relationship between potential income from RED and national-level governance indicators (Kaufmann *et al*. 2005). Data were log transformed to improve the clarity of the plot. We weighted income potential by gross domestic product (GDP) to obtain incentives from RED payments to governments relative to the size of a country's economy. Many of the countries that could in principle achieve the highest relative incomes through RED, for example, Liberia, the Democratic Republic of Congo and Myanmar, may not have sufficiently effective governance capacities to implement effect land-use policies. As a result, countries with moderate relative income benefits and higher governance indices, such as Bolivia, Nicaragua and Zambia, may be better positioned to benefit from RED in real terms. In addition, governance problems are likely to be most severe at the forest frontier, something not captured by national-level indicators (Nepstad *et al*. 2002).

There may thus be formidable challenges to realizing RED benefits from a governance perspective. Previous research has found that countries with lower governance scores tend to have higher deforestation rates and less success in conservation (Smith *et al*. 2003). Furthermore, even if lower deforestation rates are achieved, weak governance structures may make it difficult to pass on benefits to rural populations, and corrupt government agencies may show little interest in sharing benefits fairly or support bottom-up conservation initiatives, thereby diminishing the potential for human development co-benefits discussed below. However, from a more optimistic viewpoint, RED could provide the necessary incentives and funds to tackle corruption and improve governance structures which in itself may have far-reaching indirect benefits for poverty alleviation and environmental protection. For example, policy and institutional reform in Bolivia's forestry sector has led to dramatically reduced corruption in the country's forestry agency which has contributed to measurably improved forest management practices, exemplified by the country's leadership role in tropical forest certification (Ebeling & Yasue in press).

## 8. Co-benefits for biodiversity and human development

The starting point for RED is climate change mitigation, and policy makers are mainly concerned with resolving the types of issues outlined above. Nevertheless, the topic has met with great interest among organizations dealing with two other key global challenges: biodiversity loss and the situation of the rural poor. Tropical deforestation not only contributes to climate change but is also regarded as the single greatest threat to terrestrial biodiversity (Turner 1996). Conservationists broadly support RED as they anticipate biodiversity benefits in line with the funding it attracts by mitigating climate change (Laurance 2007). However, carbon markets value carbon not biodiversity and are designed to focus on the lowest cost options for generating emission reductions. They will thus favour areas with low land-use opportunity costs which may not coincide with areas of high conservation priorities. For example, global hot spots for biodiversity conservation have high land-use conversion rates (Myers *et al*. 2000) and are consequently likely to have high opportunity costs for conservation. For such areas, carbon finance alone may not be able to outweigh benefits from alternative land uses.

**Figure 4:**
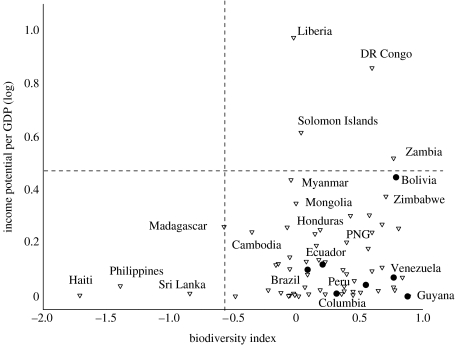
Relationship between national income potential from RED (expressed as a percentage of GDP, log transformed) and biodiversity index (variable combining proportion of threatened species, proportion of countries' eco-regions under threat and overall levels of endemism; Esty *et al*. 2005). There are no countries in the top left quadrant where highly threatened biodiversity would coincide with high relative RED income potential. Triangle, non-Amazon countries; filled circle, Amazon countries.

To evaluate the scope for synergies between RED and biodiversity conservation on a country level, we plotted a biodiversity index (Esty *et al*. 2005) for each country with net forest area loss from 1990 to 2005 against its relative income potential from RED. Countries with high index values, representing high levels of endemism and threatened species, among other things, did not have high-income potential from RED. Countries with high-income potential, such as the Democratic Republic of the Congo and Liberia, are not countries of high conservation priorities (see top right quadrant, figure 4). Moreover, on a country level, all Amazonian states are of relatively low biodiversity conservation priority, presumably because there still remain many remote, intact forest areas. There are also biodiversity-rich countries with high levels of biodiversity threats, such as Guyana and Venezuela, which will probably gain little RED carbon finance (see bottom right quadrant, figure 4). Moreover, countries in arid regions, which tend to have carbon-poor forests, are unlikely to draw significant benefits from a RED mechanism, although their forests could be of significant biodiversity value. It is important to note that there could still be significant conservation gains in particular regions *within* countries that have high-income potential from RED but score low in national biodiversity threat indices, for example, the biodiversity hot spot in the tropical Andes in Bolivia.

**Figure 5:**
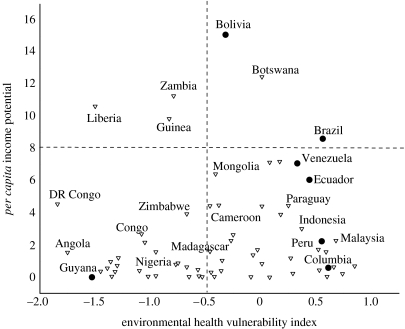
Relationship between *per capita* income potential from RED and environmental health index (variable combining death rate from infectious intestinal disease, child death rate from respiratory disease and infant mortality; Esty *et al*. 2005). This graph shows that there is no clear correlation between countries that would be priorities for improving environmental health and those with high *per capita* income potential from RED. Some of the countries where both variables coincide, e.g. Liberia, may face severe challenges to implementing RED (see figure 3). Triangle, non-Amazon countries; filled circle, Amazon countries.

There are also widespread hopes that RED will provide resources for human development and poverty relief. RED could create monetary value for natural resources which millions of the world's poor depend on for their livelihoods (White & Martin 2002). However, similarly to biodiversity co-benefits, countries with the greatest human development needs may not be countries that have high-income potentials from RED. In an analysis analogous to the above, we plotted potential *per capita* RED finance against a human development index (figure 5). The findings suggest that a pure market approach might produce few synergies between emission reductions through RED and development benefits on a national level.

In addition, the degree to which synergies between conservation and development can be realized through RED depends on how the mechanism is put into practice. Compensation payments from an international mechanism would in all likelihood accrue to national governments. Whether these payments are passed on to rural populations and communities can depend on (i) the mechanisms used to lower deforestation and (ii) the drivers of deforestation that are targeted. For example, if a country decided to rely primarily on strict law enforcement to curb deforestation, there would arguably be much fewer development benefits than if PES schemes were set up. Similarly, a government could focus its efforts on a few players, such as large agro-businesses, rather than hundreds of thousands of smallholders and forest communities. Such an approach would probably also entail lower transaction costs.

Carbon finance may be efficiently channelled towards areas and countries that are priorities for conservation and development by providing supplementary international funding for RED initiatives which specifically aim to enhance non-carbon benefits. For example, targeted conservation or development co-financing may be able to ‘tip the balance’ in favour of pro-biodiversity or pro-poor RED activities. Similarly, conservation organizations with particular experience in a site or region could provide logistical or advisory support to encourage RED activities in priority areas. In addition, some buyers in existing carbon markets are willing to preferentially buy or pay higher prices for carbon credits if these are associated with measurable conservation and development benefits (EcoSecurities 2008; Hamilton *et al*. 2007). Certification standards for verifying such impacts exist and could be effectively used to promote co-benefits through RED.

## 9. Conclusions

The current political momentum behind the RED initiative in conjunction with the dynamic negotiations for a post-2012 climate regime could link tropical forest conservation with carbon markets, the most progressive environmental finance instrument to date. Our analysis shows that there is a real potential to generate unprecedented funds for deforestation prevention; however, it also revealed a number of challenges. Achieving climatic benefits through RED will depend on several key design issues of a compensation mechanism and on striking appropriate trade-offs between environmental integrity, political and economic incentives, scientific rigour and pragmatism regarding data requirements. It will also require a careful analysis of land-use change dynamics in individual countries. Crucially, tackling deforestation and implementing RED will hinge on governance capacity in host countries. Carbon markets alone cannot be expected to overcome all these hurdles and provide sufficient incentives to governments and land users on the ground. In addition, stringent emission targets by industrialized countries are needed to create a market for RED credits and ensure that these do not replace other emission reduction efforts.

Not only may RED reduce harmful GHG emissions but this mechanism could also provide substantial co-benefits for biodiversity and human development. However, achieving these aims may require significant non-carbon-based support, and it is therefore crucial for conservationists and the development community alike to engage with climate policy makers and governments of potential RED host countries. Options for improving co-benefits range from influencing the international policy framework regarding the valuation of non-carbon benefits, to the provision of supplementary funds to implement RED measures in conservation priority areas, to supporting the choice of pro-poor land-use policies in host countries. Several decades of experience already exist in tackling tropical deforestation, identifying and protecting biodiversity-rich areas, and promoting rural development (Kramer *et al*. 1997; Peres & Zimmerman 2001) and there may now be a chance to implement many lessons in a more supportive political environment and with more financial resources. While it is important not to raise unrealistic expectations about RED, overcoming the current challenges offers a historic opportunity of saving the remaining tropical forests while mitigating climate change.

## Figures and Tables

**Table 1:** Deforestation in Amazon countries (member states of ACTO) 1990–2005. (Source: FAO (2001, 2006)).

country	average annual change in forest area	

	absolute (ha)	relative (%)
Bolivia	−270 000	−0.45
Brazil	−2 821 670	−0.55
Colombia	−47 670	−0.10
Ecuador	−198 000	−1.60
Guyana	(0)	(0.00)
Peru	−94 000	−0.10
Suriname	(0)	(0.00)
Venezuela	−288 000	−0.60
total	−3 719 340	−0.20

## Supplementary Material

Potential economic, conservation and development benefits of carbon trading in Non-Annex 1 countries For column headings C, carbon; PI, potential annual income; GDP, gross domestic product
