# Supplementary material for: Generating carbon finance through avoided deforestation and its potential to create climatic, conservation and human development benefits
Source: Philos Trans R Soc Lond B Biol Sci. 2008 Feb 11;363(1498):1917–24. doi: 10.1098/rstb.2007.0029 (PMC2610211; doi:10.1098/rstb.2007.0029)
Supplement: Potential economic, conservation and development benefits of carbon trading in Non-Annex 1 countries — For column headings C, carbon; PI, potential annual income; GDP, gross domestic product [file rstb20070029s06.pdf]

Appendix 1. Potential economic, conservation and development benefits of carbon trading in Non-Annex 1 countries. For column headings C = carbon, PI = potential annual income, GDP = Gross Domestic Product

Country	Forest statistics				Economic incentive (annually)			Potential co-benefit indicators		Governance challenge
	% of total land area forested	Area (1000 ha)	% Avg. annual change (1990-2005)	Avg. C density (tC/ha)	*PI from C trading(€ million)	*PI as % of 2005 GDP	PI per capita (€)	**Biodiversity	**Environmental Health	***Governance
Afghanistan	1.33	867	-2.80	13.5	64.4	0.04	0.07			-1.57
Algeria	1.00	2,277	1.50	37.5	470.1	0.01	0.20	-0.08	-0.33	-0.61
Angola	47.40	59,104	-0.20	27.0	8784.9	0.08	1.55	0.77	-1.75	-1.23
Argentina	11.88	33,021	-0.40	34.0	6180.5	0.02	0.70	0.10	0.85	-0.58
Armenia	9.50	283	-1.40	33.0	51.4	0.02	0.24	-0.02	0.29	-0.56
Azerbaijan	11.33	936	0.00	52.5	270.5	0.00	0.00	-0.13	-1.17	-0.95
Bahamas	133.07	515	0.00	50.0	141.8	0.00	0.00			1.32
Bangladesh	6.05	871	-0.23	19.5	93.5	0.00	0.00	-0.48	0.08	-0.98
Barbados	4.65	2	0.67	50.0	0.6	0.00	0.00			1.01
Belize	71.99	1,653	0.00	105.5	960.0	0.00	0.00			0.09
Benin	20.88	2,351	-2.30	97.5	1261.9	1.01	4.44	0.43	-0.46	-0.41
Bhutan	67.98	3,195	0.30	89.0	1565.4	0.85	2.36	-0.12	-0.47	0.48
Bolivia	53.47	58,740	-0.45	91.5	29587.8	1.81	15.13	0.79	-0.32	-0.67
Botswana	21.10	11,943	-0.95	31.5	2071.0	0.28	12.48	0.68	0.01	0.80
Brazil	56.10	477,698	-0.55	104.5	274806.5	0.26	8.63	0.09	0.56	-0.18
Brunei	48.18	278	-0.75	102.5	156.9	0.02	2.97			0.40
Burkina Faso	29.00	6,794	-0.30	8.0	299.2	0.02	0.08	0.32	-0.95	-0.49
Burundi	5.46	152	-4.45	93.5	78.2	0.74	0.57	0.60	-1.39	-1.33
Cambodia	57.71	10,447	-1.55	34.5	1984.1	0.75	2.28	-0.35	-0.27	-0.98
Cameroon	44.68	21,245	-0.95	65.5	7660.5	0.60	4.49	0.40	-0.38	-0.89
Cape Verde	20.84	84	2.00	63.5	29.4	0.09	1.66			0.29
Central African Republic	36.53	22,755	-0.10	56.5	7077.5	0.87	2.17	0.68	-1.05	-1.40
Chad	9.50	11,921	-0.65	8.0	525.0	0.08	0.35	0.07	-1.32	-1.15
Chile	21.31	16,121	0.40	134.0	11892.0	0.05	2.61	-0.50	0.84	1.30
China	20.56	197,290	1.70	30.5	33125.5	0.03	0.34	-0.04	0.60	-0.49
Colombia	53.32	60,728	-0.10	98.0	32762.1	0.03	0.59	0.32	0.61	-0.43
Comoros	2.90	5	-5.70	32.5	0.9	0.25	1.04			-1.09

**Congo**	65.70	22,471	-0.10	106.5	13174.4	0.18	2.69	0.84	-1.08	-1.10
**Congo, DR**	56.98	133,610	-0.30	112.5	82746.3	6.24	4.56	0.60	-1.84	-1.53
**Costa Rica**	46.79	2,391	-0.35	110.0	1447.9	0.05	1.73	0.38	0.52	0.68
**Côte d'Ivoire**	32.27	10,405	0.10	65.0	3723.2	0.03	0.250	0.01	-0.69	-1.22
**Djibouti**	0.20	6	0.00	23.0	0.8	0.00	0.000			-0.78
**Dominican Republic**	28.24	1,376	0.00	26.5	200.7	0.00	0.000	-1.20	0.00	-0.52
**Ecuador**	38.27	10,853	-1.60	75.5	4510.8	0.32	6.074	0.21	0.44	-0.73
**Egypt**	0.10	67	2.80	53.0	19.5	0.00	0.007	0.02	-1.84	-0.12
**El Salvador**	14.16	298	-1.60	101.0	165.7	0.02	0.407	0.36	-0.36	-0.37
**Equatorial Guinea**	58.18	1,632	-0.85	79.0	709.7	0.12	12.078			-1.35
**Eritrea**	15.40	1,554	-0.30	16.0	136.9	0.05	0.074			-0.71
**Ethiopia**	11.90	13,000	-1.05	39.5	2826.8	0.35	0.410	0.08	-1.10	-0.93
**Fiji**	54.73	1,000	0.10	32.0	176.2	0.01	0.259			-0.17
**Gabon**	81.35	21,775	-0.05	68.5	8211.2	0.05	2.646	0.78	-0.25	-0.55
**Gambia**	41.68	471	0.40	11.0	28.5	0.03	0.074	0.59	-0.74	-0.47
**Georgia**	39.60	2,760	0.00	48.5	736.9	0.00	0.000	-0.27	0.42	-0.89
**Ghana**	23.13	5,517	-2.00	44.0	1336.3	0.37	1.383	0.18	-0.07	-0.17
**Guatemala**	36.16	3,938	-1.25	185.5	4021.4	0.26	4.428	0.37	0.01	-0.85
**Guinea**	27.35	6,724	-0.60	57.0	2109.9	0.55	9.865	0.16	-0.83	-0.95
**Guinea-Bissau**	57.36	2,072	-0.45	10.0	114.1	0.25	0.057	0.60	-1.02	-0.99
**Guyana**	70.26	15,104	0.00	126.5	10518.2	0.00	0.000	0.88	-1.53	-0.42
**Haiti**	3.78	105	-0.95	50.5	29.2	0.01	0.033	-1.71	-0.65	-1.58
**Honduras**	41.47	4,648	-3.05	52.5	1343.3	0.82	7.206	-0.07	0.17	-0.66
**India**	20.59	67,701	0.37	36.5	13603.3	0.01	0.045	-0.62	0.08	-0.20
**Indonesia**	46.46	88,495	-1.85	68.0	33127.2	0.33	3.022	-0.14	0.37	-0.91
**Iran**	6.72	11,075	0.00	74.5	4542.1	0.00	0.000	-0.08	0.08	-0.71
**Jamaica**	31.30	339	0.00	85.5	159.6	0.00	0.068	-1.20	0.66	-0.42
**Jordan**	0.93	83	0.00	18.5	8.5	0.00	0.000	0.14	0.22	0.33
**Kazakhstan**	1.22	3,337	-0.20	9.0	165.3	0.00	0.020	0.02	-0.09	-1.04
**Kenya**	6.20	3,522	-0.30	24.0	465.3	0.01	0.047	0.45	-0.42	-0.94
**Kiribati**	2.74	2	0.00	32.0	0.4	0.00	0.000			0.12
**Korea**	63.12	6,265	-0.10	18.0	620.8	0.00				0.42
**Kuwait**	0.34	6	3.10	10.5	0.3	0.00	0.005	-0.66	0.08	0.68
**Kyrgyzstan**	4.35	869	0.30	10.0	47.8	0.01	0.021	0.06	-0.44	-0.98
**Laos**	68.17	16,142	-0.50	15.5	1377.4	0.31	1.045	-0.16	-0.35	-1.21
**Lebanon**	13.08	136	0.80	11.0	8.2	0.00	0.016	-0.07	0.11	-0.42

**Lesotho**	0.30	8	3.05	17.0	0.7	0.00	0.009			-0.04
**Liberia**	28.32	3,154	-1.70	98.0	1701.6	8.43	10.641	-0.02	-1.50	-1.31
**Libya**	0.10	217	0.00	10.0	11.9	0.00	0.000	0.22	0.02	-0.78
**Madagascar**	22.10	12,838	-0.40	97.0	6855.3	0.83	1.613	-0.57	-0.46	-0.23
**Malawi**	36.20	3,402	-0.90	71.5	1339.1	0.80	0.978	0.81	-1.36	-0.56
**Malaysia**	63.35	20,890	-0.55	102.5	11787.4	0.05	2.283	-0.22	0.68	0.41
**Mali**	10.30	12,572	-0.75	15.5	1072.7	0.21	0.731	0.09	-1.30	-0.43
**Mauritania**	0.30	267	-3.05	3.0	4.4	0.01	0.052	-0.01	-1.35	-0.30
**Mexico**	32.80	64,238	-0.45	27.0	9548.0	0.01	0.441	-0.05	0.53	-0.27
**Mongolia**	0.00	0	-0.01	40.0	0.0	1.24	6.427	0.00	-0.41	-0.17
**Morocco**	9.80	4,364	0.15	20.5	492.5	0.00	0.017	-0.31	0.18	-0.04
**Mozambique**	24.60	19,262	-0.30	27.5	2916.0	0.14	0.369	0.46	-1.45	-0.70
**Myanmar**	47.62	32,222	-1.35	28.5	5055.4	1.74	1.715	-0.04	-0.03	-1.56
**Namibia**	9.30	7,661	-0.90	6.0	253.0	0.05	1.185	0.65	0.27	0.20
**Nepal**	24.70	3,636	-1.75	54.5	1090.9	0.40	0.838	-0.05	0.01	-0.72
**Nicaragua**	39.92	5,189	-1.45	80.5	2299.5	1.02	7.160	0.58	0.08	-0.50
**Niger**	1.00	1,266	-2.35	2.0	13.9	0.02	0.040	0.23	-1.53	-0.90
**Nigeria**	12.00	11,089	-3.00	92.0	5616.1	0.27	1.575	-0.05	-0.95	-1.28
**Oman**	0.01	2	0.00	8.5	0.1	0.00	0.000	-0.27	-0.17	0.88
**Pakistan**	2.39	1,902	-1.95	13.5	141.4	0.00	0.019	-0.06	-0.53	-0.83
**Panama**	56.86	4,294	-0.15	161.0	3805.8	0.04	1.574	0.45	0.59	-0.05
**Papua New Guinea**	63.60	29,437	-0.50	29.0	4699.5	0.72	3.913	0.15	0.18	-0.86
**Paraguay**	45.42	18,475	-0.90	29.5	3000.3	0.51	4.468	0.57	0.25	-1.04
**Peru**	53.49	68,742	-0.10	122.5	46357.0	0.10	2.240	0.55	0.55	-0.49
**Philippines**	23.87	7,162	-2.45	57.0	2247.3	0.09	0.796	-1.39	-0.77	-0.59
**Puerto Rico**	45.59	408	0.07	50.0	112.3	0.00	0.019			0.81
**Rwanda**	18.22	480	3.85	93.5	247.1	0.34	0.587	0.48	-0.94	-0.63
**Samoa**	60.21	171	1.40	32.0	30.1	0.18	2.655			0.34
**São Tomé and Príncipe**	28.13	27	0.00	58.0	8.6	0.00	0.000			-0.61
**Saudi Arabia**	1.27	2,728	0.00	6.0	90.1	0.00	0.000	-0.21	-0.23	0.18
**Senegal**	44.09	8,673	-0.50	15.0	716.2	0.06	0.305	0.19	-0.45	-0.30
**Seychelles**	88.90	40	0.00	24.5	5.4	0.00	0.000	-0.11		-0.08
**Sierra Leone**	38.39	2,754	-0.70	69.5	1053.7	0.78	1.210	0.19	-1.29	-0.99
**Singapore**	2.94	2	0.00	102.5	1.1	0.00	0.000	0.07		2.13
**Solomon Islands**	75.16	2,172	-1.60	32.0	382.6	3.14	12.755	0.04		-1.19
**South Africa**	7.60	9,203	0.00	40.5	2051.8	0.00	0.000	-1.32	0.09	0.40

**Sri Lanka**	29.46	1,933	-1.35	29.5	313.9	0.02	0.219	-0.84	0.74	-0.10
**St. Kitts and Nevis**	13.89	5	0.00	50.0	1.4	0.00				0.53
**St. Lucia**	27.42	17	0.00	99.0	9.3	0.00				0.52
**St. Vincent and the Grenadines**	28.21	11	0.80	86.5	5.2	0.02				0.55
**Sudan**	28.40	67,546	-0.80	6.0	2231.0	0.09	0.472	0.38	-0.57	-1.45
**Suriname**	90.50	14,776	0.00	126.5	10289.7	0.00	0.000			0.05
**Swaziland**	31.50	541	0.90	57.5	171.2	0.08	1.393			-0.95
**Syria**	2.49	461	1.40	14.0	35.5	0.00	0.024	0.36	0.03	-0.57
**Tajikistan**	2.88	410	0.00	5.0	11.3	0.00	0.001	0.07	-2.17	-1.15
**Tanzania**	39.90	35,257	-1.05	14.5	2814.3	0.35	0.878	0.23	-0.75	-0.53
**Thailand**	28.30	14,520	-0.55	77.5	6194.8	0.03	0.636	-0.12	0.66	-0.15
**Togo**	6.80	386	-3.95	32.0	68.0	0.21	0.635	0.40	-0.67	-0.97
**Tonga**	5.33	4	0.00	64.5	1.4	0.00	0.000	0.89		-0.40
**Trinidad and Tobago**	44.05	226	-0.25	13.5	16.8	0.00	0.042	0.89	0.60	0.09
**Tunisia**	6.80	1,056	3.00	1.5	8.7	0.00	0.022	0.00	0.19	0.27
**Turkmenistan**	8.46	4,127	0.00	81.5	1851.6	0.00	0.000	0.19	-2.64	-1.39
**Uganda**	18.40	3,627	-2.05	5.0	99.8	0.03	0.078	0.51	-0.56	-0.75
**United Arab Emirates**	3.73	312	1.25	30.0	51.5	0.00	0.166	-0.28	0.44	1.04
**Uruguay**	8.55	1,506	2.90	29.5	244.6	0.05	1.877	-0.18	0.85	0.46
**Uzbekistan**	7.36	3,295	0.50	5.0	90.7	0.01	0.017	0.30	-0.98	-1.26
**Vanuatu**	36.10	440	0.00	32.0	77.5	0.00	0.000			-0.30
**Venezuela**	52.31	47,713	-0.60	116.5	30599.9	0.18	7.097	0.77	0.33	-1.02
**Vietnam**	38.99	12,931	2.15	33.0	2349.1	0.11	0.512	-0.35	0.34	-0.67
**Yemen**	1.04	549	0.00	9.5	28.7	0.00	0.000	-0.13	-1.15	-0.98
**Zambia**	57.10	42,452	-0.95	52.0	12152.3	2.31	11.285	0.77	-0.79	-0.64
**Zimbabwe**	45.30	17,540	-1.60	28.0	2703.6	1.38	3.943	0.71	-0.67	-1.27

***Calculated based on scenario of 10 % deforestation reduction and € 15 / tCO2**

****Environmental Sustainability Index (Esty *et al.* 2005)**

*****Avg value of two World Bank governance indicators (adapted from Kaufmann *et al*. 2005)**
